# Island building in the South China Sea: detection of turbidity plumes and artificial islands using Landsat and MODIS data

**DOI:** 10.1038/srep33194

**Published:** 2016-09-15

**Authors:** Brian B. Barnes, Chuanmin Hu

**Affiliations:** 1 College of Marine Science, University of South Florida, St Petersburg, FL, USA

## Abstract

The South China Sea is currently in a state of intense geopolitical conflict, with six countries claiming sovereignty over some or all of the area. Recently, several countries have carried out island building projects in the Spratly Islands, converting portions of coral reefs into artificial islands. Aerial photography and high resolution satellites can capture snapshots of this construction, but such data are lacking in temporal resolution and spatial scope. In contrast, lower resolution satellite sensors with regular repeat sampling allow for more rigorous assessment and monitoring of changes to the reefs and surrounding areas. Using Landsat-8 data at ≥15-m resolution, we estimated that over 15 km^2^ of submerged coral reef area was converted to artificial islands between June 2013 and December 2015, mostly by China. MODIS data at ≥250-m resolution were used to locate previously underreported island building activities, as well as to assess resulting in-water turbidity plumes. The combined spatial extent of observed turbidity plumes for island building activities at Mischief, Subi, and Fiery Cross Reefs was over 4,300 km^2^, although nearly 40% of this area was only affected once. Together, these activities represent widespread damage to coral ecosystems through physical burial as well as indirect turbidity effects.

The Spratly Islands are a large archipelago of coral reefs and islands in the South China Sea (SCS, Fig. 1). Although no inhabitants are native to these islands, their strategic location has large implications for sovereignty issues, especially for sea-lane security. As such Brunei, China, Malaysia, the Philippines, Taiwan (or Republic of China), and Vietnam each claim all or part of the archipelago and associated waters, creating intense geopolitical conflicts. Fisheries, natural gas, and crude oil also factor into the disputes, although the importance of the latter is often overstated^1^. One means which the bordering countries have used to bolster their sovereignty claims is to occupy the islands, which has been ongoing for many decades in the SCS^2^.

As a consequence of the geopolitical tensions, there is sustained interest in detecting and monitoring changes in the region. Among such changes, several countries (especially China and Vietnam) have recently embarked on island building activities in the region, converting all or part of several submerged coral reefs into artificial islands (Fig. 1). For China, such recent projects include construction on Mischief Reef, Subi Reef, Fiery Cross Reef, Caurteron Reef, Gaven Reef, Hughes Reef, and Johnson South Reef (Figs 2 and 3). Recent construction by Vietnam has occurred on Spratly Island, Sin Cowe Island, Cornwallis South Reef, West Reef, Sand Cay, Pearson Reef, Central Reef, Southwest Cay, Namyit Island, and Grierson Reef (Fig. 4). In addition, Taiwan has recently expanded the port on Itu Aba Island, while the Philippines and Malaysia have conducted older island building projects on Thitu Island and Swallow Reef, respectively. Beyond the Spratly Islands, several other artificial island projects in the SCS (particularly within the Paracel Islands) are underway. Many of these projects have been extensively reported by the international media, and have also been captured in aerial photography as well as commercial satellite imagery^3^,^4^. While these methods have provided extremely high resolution snapshots of specific projects, both methods are costly and thus lacking in temporal resolution (especially prior to the start of construction). As a consequence, quantitative assessment of these island building activities in space and time has been lacking. Furthermore, due to the sensitive geopolitical situation, collection of aerial photography may be viewed as inflammatory.

In contrast, publically available data from lower-resolution satellites provide regular observations of this region, which might also be used to more robustly capture these changes (e.g. ref. 5). In particular, despite primarily land applications, the Operational Land Imager (OLI) onboard the United States Geological Survey (USGS) satellite Landsat-8 has been providing reflectance data for the Spratly Islands region since mid-2013. OLI data are collected at 30 m spatial resolution (a pan-sharpening band has 15 m resolution) with 16-day repeat sampling frequency. Also, the Moderate Resolution Imaging Spectroradiometer (MODIS) onboard the satellite Aqua (MODIS/A) has provided global 250–1000 m resolution reflectance data at nearly daily temporal resolution since 2002.

Compared to aerial photography and commercial satellite data, the high temporal resolution and wide swath of MODIS/A and OLI increase the ability to detect and monitor island building activities and resulting environmental changes. In particular, the regular observations preceding the start of many of these construction projects enhance the capacity to place these events in historical context, as baseline conditions can be established from these historical data. As such, the overall goal of this work was to use OLI and MODIS/A data to identify and assess changes in the Spratly Islands region of the South China Sea, with specific objectives to (1) identify reef locations converted to artificial islands and document their size, and (2) quantify effects of island building activities on water turbidity.

## Methods

### MODIS data acquisition and processing

MODIS/A Level-0 data from 2013–2016 covering the SCS were downloaded from the National Aeronautics and Space Administration (NASA) ocean color archives (oceancolor.gsfc.nasa.gov). Individual satellite passes were processed using SeaDAS^6^ (version 7.2) to 250 m resolution Level-2 products including chlor_a (chlorophyll-a concentration, mg m^−3^), multispectral remote sensing reflectance (*R*_*rs*_, sr^−1^), multispectral calibrated at-sensor total radiance (*L*_*t*_; W m^−2^ um^−1^ sr^−1^), multispectral Rayleigh radiance (*L*_*r*_; W m^−2^ um^−1^ sr^−1^), and Level-2 processing flags (l2_flags). Note that for many of these products, this processing included interpolation from native 500 m or 1 km spatial resolution. The lower-level MODIS/A data were additionally processed using the MOD35 algorithm^7^ (version 3.1) for cloud detection at 250 m resolution. All of these products were mapped at 250 m resolution (using SeaDAS/Beam) to an equidistant cylindrical projection with bounds 8.5°–11.5N, 112–116°E. From this, *R*_*rc*_ for each MODIS band was calculated as


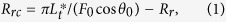


where 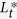 is total at-sensor radiance adjusted for ozone and gaseous absorption, *F*_*0*_ is the extraterrestrial solar irradiance, *Θ*_*0*_ is the solar zenith angle, and *R*_*r*_ is Rayleigh reflectance estimated from SeaDAS *L*_*r*_^8^,^9^. True-color RGB composites were created using *R*_*rc*_ data at the 645, 555, and 469 nm bands (each scaled from 0 to 0.15). As the former has native 250 m resolution and the latter two have 500 m resolution, the red/green and red / blue ratios were used to sharpen the green and blue inputs toward the final RGB composite at 250-m resolution.

### MODIS/A detection of shallow water and artificial islands

The absorption coefficient for pure water in the red and near infrared (NIR) wavelengths is very high^10^,^11^, meaning the *R*_*rs*_ or *R*_*rc*_ for clear, deep water targets in these wavelengths is low. In contrast, *R*_*rs*_(NIR) is much higher for land and cloud targets, and *R*_*rs*_ in the red bands can also be high in response to in-water turbidity or shallow-water benthic contributions to the *R*_*rs*_ signal. As such, and in response to uncertainties in reef mapping in Burke *et al*.^12^, we identified shallow waters using the MODIS/A *R*_*rs*_(645) product. Specifically, any pixels for which *R*_*rs*_(645) was found to be >0.005 sr^−1^ in at least 20 satellite passes in 2013 were identified as shallow waters. Similarly, we used changes in MODIS/A *R*_*rc*_(859) to identify newly created artificial islands at 250 m spatial resolution. This product reliably differentiated land from water targets, but improper cloud masking severely limited robustness of results. We thus measured the slope of *R*_*rc*_(859) from 2013–2016 for each pixel location. The highest values in this metric were subsequently examined using OLI data.

### OLI data acquisition and processing

**Figure 1:**
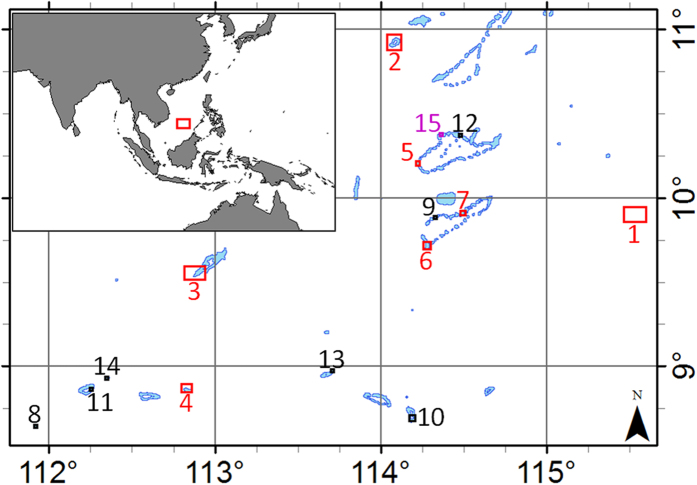
Coral reefs in the Spratly Islands region of the South China Sea (blue; Burke *et al*.^12^). Reefs with significant development since 2013 indicated by numbered squares (see Figs 2, 3, 4). Red (1–7), black (8–14), and purple (15) squares denote development by China, Vietnam, and Taiwan, respectively. Note that Mischief Reef (square #1) is not well represented in Burke *et al*.^12^. The location of the region is annotated in the inset map. This figure was created using ArcMap (version 10.2).

**Figure 2:**
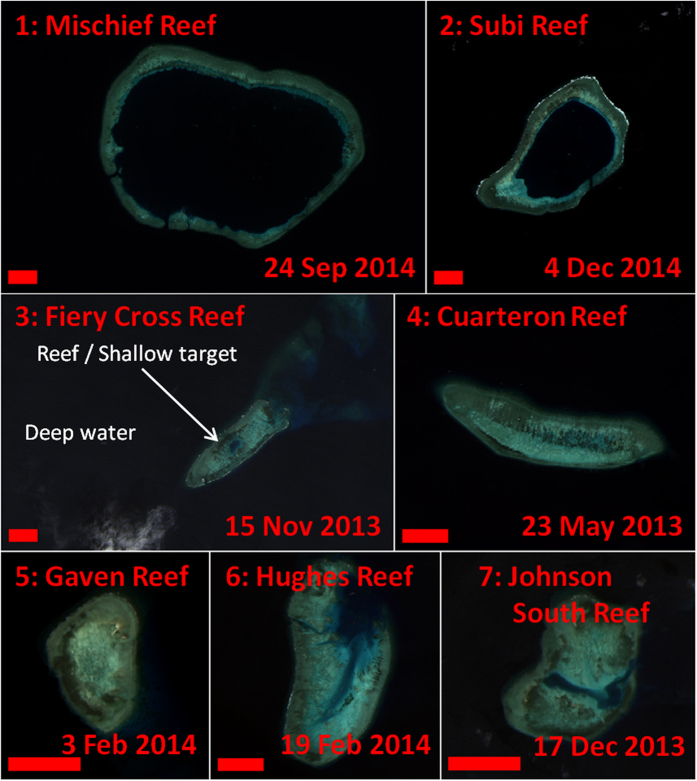
OLI imagery of reefs (1–7) outlined in Fig. 1. Images were taken prior to commencement of island building. Red bar length indicates 1 km. Green, brown, and turquoise pixels indicate coral reefs or other shallow targets. Black and blue pixels indicate clear deep water. These images were created using data from the USGS (http://earthexplorer.usgs.gov/) and processed using Acolite (version 20150701.1) as detailed in the methods section.

**Figure 3:**
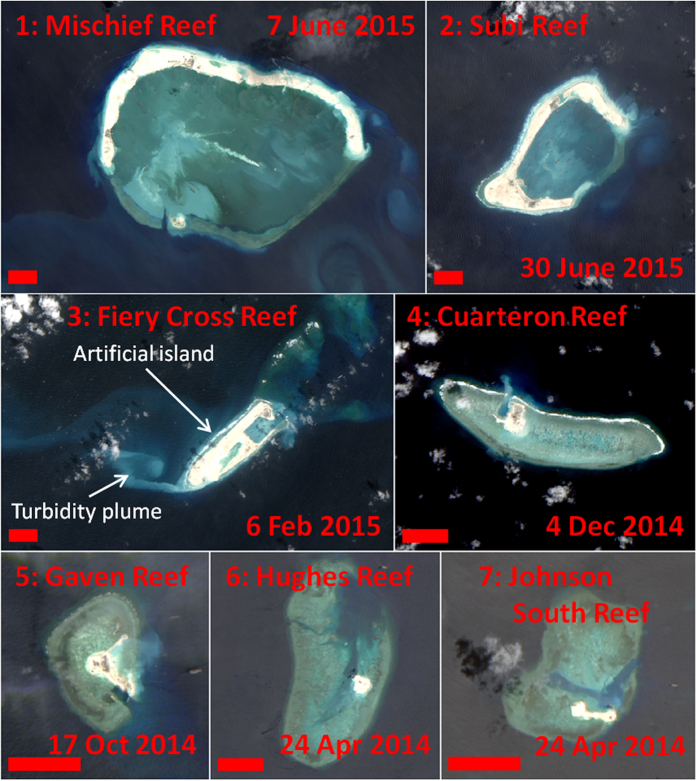
OLI imagery of reefs (1–7) outlined in Fig. 1. These reefs are under various stages of island building by China. Note this is the same color stretch as used in Fig. 2, but some images are brightened by sunglint or atmospheric haze. New artificial islands appear bright white, while turbidity plumes appear white or cyan over deep-water targets (see Fig. 2). These images were created using data from the USGS (http://earthexplorer.usgs.gov/) and processed using Acolite (version 20150701.1) as detailed in the methods section.

**Figure 4:**
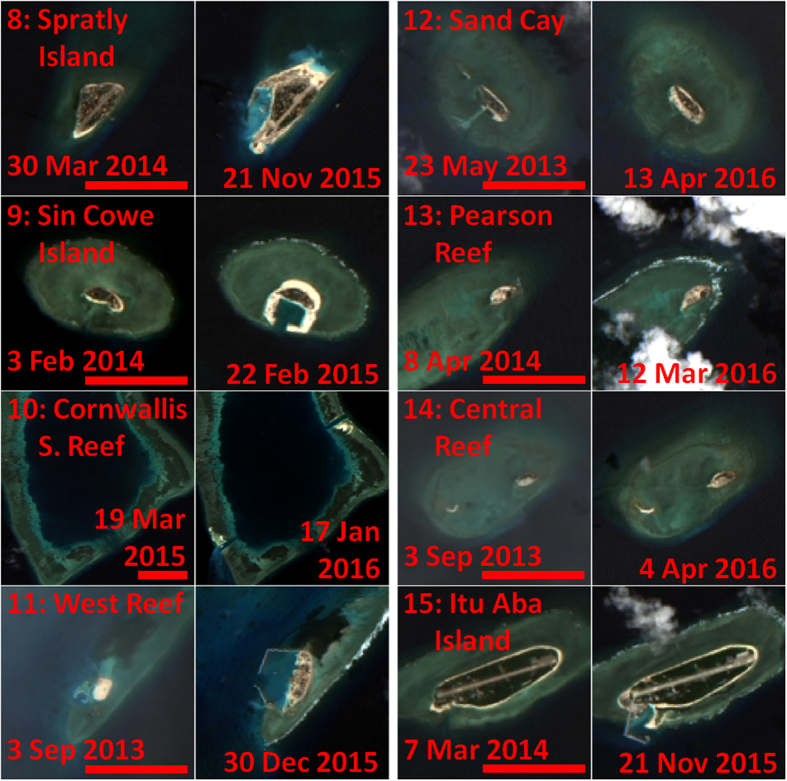
OLI imagery of reefs (8–15) outlined in Fig. 1. Images in columns 1 and 3 were taken prior to commencement of recent island building (or near start of OLI time series for projects which began prior to OLI). Images in columns 2 and 4 were taken after completion of recent island building (or near the time of this writing for ongoing projects). Red bar length indicates 1 km. These images were created using data from the USGS (http://earthexplorer.usgs.gov/) and processed using Acolite (version 20150701.1) as detailed in the methods section.

All Landsat-8 OLI data for six locations (path/rows 119/053, 119/054, 120/052, 120/053, 120/054, 121/054) were downloaded from the USGS via the Landsat Amazon Web Service. These data were processed to Rayleigh-corrected reflectance (*R*_*rc*_; dimensionless) using SWIR-based atmospheric correction^13^ in Acolite (version 20150701.1). True-color RGB composites were created with a scaling of 0 to 0.5 for each of the 655, 561, and 483 nm bands, which were sharpened using the pan-chromatic band. The RGB images were subset to isolate reefs where recent island building activity had been reported^3^,^4^ or detected using MODIS data (Fig. 1, see section 2.2). These reef-specific true-color RGB composites were georeferenced and imported into ArcMap (version 10.2), whereby changes in areal extent of reefs and artificial islands were quantified (see Figs 2, 3, 4).

### MODIS/A turbidity plume detection

**Figure 5:**
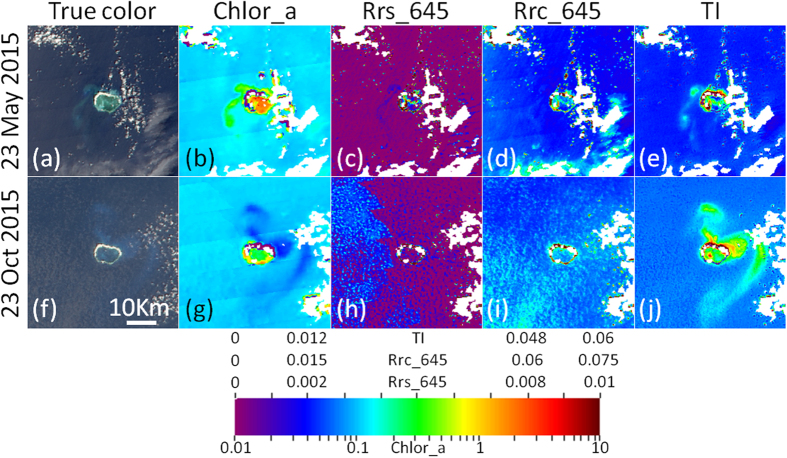
True color RGB composites (first column) covering Mischief Reef region indicate turbidity plumes visible from MODIS imagery on 23 May 2015 (top row) and 23 Oct 2015 (bottom row). Standard products of chlor_a (mg m^−3^, second column), *R*_*rs*_(645) (sr^−1^, third column) and *R*_*rc*_(645) (dimensionless, fourth column) show difficulty in capturing plumes. Turbidity index (dimensionless, fifth column) developed in this is work shown for comparison. Note that a uniform (yet non-standard) colorbar was applied to all products for simplicity, and all products were masked for clouds (white color) using the CLDICE, LAND, and HILT Level-2 processing flags (Patt *et al*.^17^). These images were created using data downloaded from the NASA (oceancolor.gsfc.nasa.gov) and processed using SeaDAS (version 7.2) as detailed in the methods section.

Barnes *et al*.^14^ noted increased capacity to monitor turbidity plumes using a combination of true color imagery and reflectance data. As such, we visually assessed the ability of the various ocean color products (chlor_a, *R*_*rs*_, *R*_*rc*_) in capturing island building activities (especially turbidity plumes) that were visible in the MODIS/A true color RGB composites (Fig. 5). While the chlor_a product generally identified plumes, the sign of the chlor_a anomaly varied between images (e.g., high in Fig. 5b, low in Fig. 5g). Traditionally, elevated red band reflectance has been used as a proxy for in-water turbidity^15^. However, neither *R*_*rs*_(645) nor *R*_*rc*_(645) were effective at capturing the turbidity plumes of interest, owing to speckling noise and effects from moderate sunglint contamination (e.g., Fig. 5h). Although only data from the 645 nm band are shown in Fig. 5, the other MODIS/A red bands showed similar difficulty in identifying these turbidity plumes. Due to these errors in traditional approaches, we examined the spectral shape of turbidity features to those of clear waters (i.e., with low turbidity). From this, we developed a simple subtraction algorithm (termed ‘Turbidity Index’ or TI), as the difference *R*_*rc*_(469)–*R*_*rc*_(645), which allowed for easy detection of turbidity plumes (Fig. 5e,j). Note that despite the name ‘Turbidity Index’, this metric is simply a band difference that is sensitive to a variety of targets, including clouds, optically shallow water, and artificial islands.

Unlike *R*_*rs*_ and *R*_*rc*_, these TI data were somewhat resilient to moderate glint contamination. However, due to the spectral shape of sunglint, TI data still varied proportionally to the glint intensity. Using the approach of Hu^16^, we rescaled *R*_*rc*_ at 645 and 469 nm to remove the effects of glint contamination. Specifically, for pixels with R_rc_(859) >0.01, R_rc_ for these two bands were modified such that


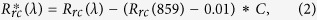


where *C* is the scaling coefficient (0.67 and 0.94 for the 469 and 645 bands, respectively). In these glint-tolerant TI maps, determination of low quality pixels using the standard L2 processing flags was deemed too restrictive (especially those flags identifying high reflectance or glint; e.g., HILT, HIGLINT, and MODGLINT; see ref. 17). As such, a cloudmask based on the 250 m MOD35 cloud product was implemented. Any pixel identified by this mask was dilated by 500 m to generate the final cloudmask that was applied to all data.

**Figure 6:**
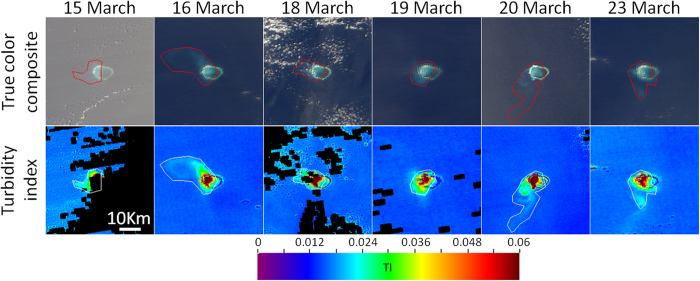
Time series of true color RGB composites (first row) covering Mischief Reef and corresponding turbidity index product (second row), with manually delineated turbidity plumes outlined in red and white, respectively. All images were collected in 2015. These images were created using data downloaded from the NASA (oceancolor.gsfc.nasa.gov) and processed using SeaDAS (version 7.2) as detailed in the methods section.

Turbidity plumes resulting from the island building activities were identified for the Mischief, Subi, and Fiery Cross Reefs. To do so, daily MODIS/A true color RGB composites and corresponding TI products were viewed simultaneously in a custom IDL-based graphical user interface (GUI). Within this GUI, manual delineations were drawn to encompass any plume features visible in either the RGB composites or TI imagery (see Fig. 6). The number of pixels included in these delineations was quantified and multiplied by 1/16 to estimate the plume areal extent in km^2^.

## Results

### Artificial island detection using MODIS

**Figure 7:**
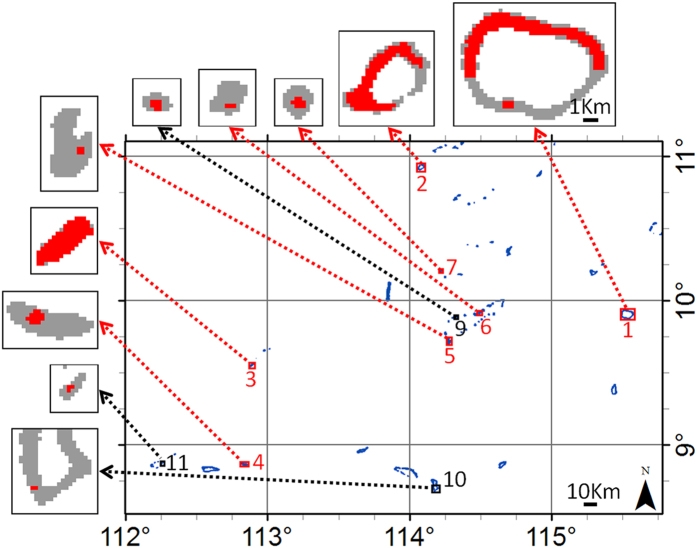
MODIS/A detection of island building in the SCS. Red in insets indicate MODIS-detected artificial island construction. Red subset demarcations denote development by China, while black indicates Vietnam development. Shallow targets (blue pixels in large image, grey in insets) were estimated as pixels with *R*_*rs*_(645) >0.005 sr^−1^ in at least 20 satellite passes in 2013. All insets have the same scale. The insets contain all pixels identified as new artificial islands in the scene.

If a location of interest was known (e.g., from media coverage), visual identification of island building activities with OLI data was straightforward (Figs 2, 3, 4). However, finding such activities within the vast South China Sea by visual examination of OLI data alone was not practical, due to the high spatial resolution (and therefore small footprint) and the persistent clouds. As such, the slope of MODIS/A *R*_*rc*_(859) at each pixel was used to identify the pixels which had likely changed from water to land targets, with higher slope values indicating artificial island creation. In this manner, many of the island building projects which had been extensively covered by the international media (e.g., refs 3 and 4) were identified, as were some that were only sparsely reported (e.g., Cornwallis South Reef). The few exceptions were projects which started prior to the beginning of the MODIS/A time series used in the metric (e.g., Sand Cay and Central Reef) or projects which represented only small changes in land area (e.g., Sand Cay, Pearson Reef, Central Reef, and Itu Aba Island; see Table 1). Note that Spratly Island lies outside the bounds of the MODIS/A data used in this analysis. Through a sensitivity analysis, we found a threshold for *R*_*rc*_(859) slope of 0.013 sr^−1^ y^−1^ could identify new artificial islands with no false positives (Fig. 7). Below this threshold, errors in cloudmasking affecting the slope metric lowered this accuracy.

### Island building detection using OLI

Using Landsat-8 OLI data, island building activities and turbidity plumes were observed for nearly all of the locations reported in the media or identified using MODIS/A (Figs 2, 3, 4), with the only exceptions being projects completed prior to the OLI time series (specifically, Southwest Cay, Namyit Island, Grierson Reef, Swallow Reef, and Thitu Island). Of the remaining island building activities studied, three of Vietnam’s projects commenced prior to the OLI dataset beginning in mid-2013 (West Reef, Sand Cay, and Central Reef), while 5 Chinese and 2 Vietnamese projects began in 2014 (for 2015, these numbers are 2 and 3, respectively). All but two of Vietnam’s projects (as well as Taiwan’s construction on Itu Aba Island) observed in the OLI time series were additions to already existing islands, while all of the Chinese projects represented entirely new islands.

**Figure 8:**
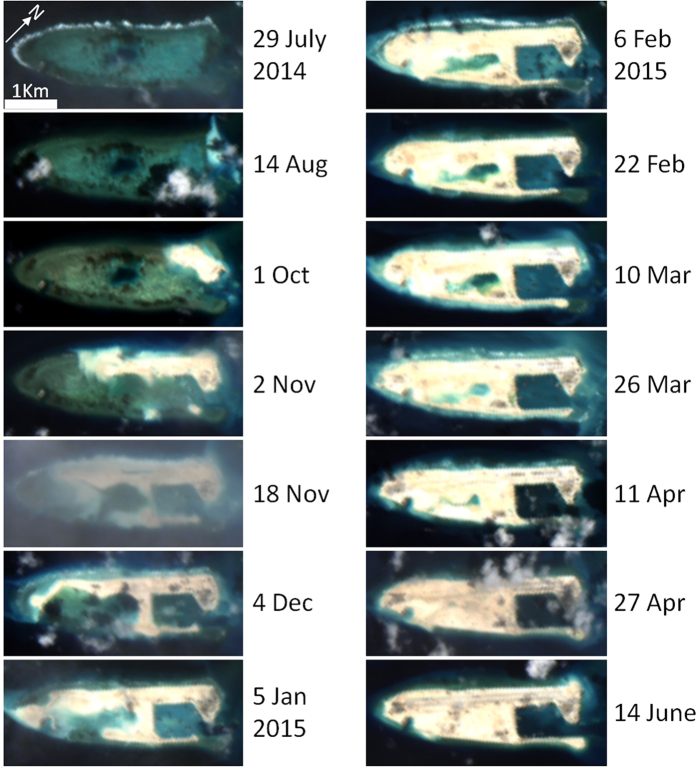
Time series of island building for Fiery Cross Reef from Landsat-8 OLI. These images were created using data from the USGS (http://earthexplorer.usgs.gov/) and processed using Acolite (version 20150701.1) as detailed in the methods section.

The final artificial island sizes for three of the Chinese projects (Mischief, Subi, and Fiery Cross Reefs) were quite large (>3 km^2^), while the remaining artificial islands were substantially smaller (<0.25 km^2^). Individual projects ranged in duration from 3 months to over two years, while Chinese projects were generally completed much more quickly than similarly sized Vietnamese or Taiwanese projects. Indeed, the rate of island building (i.e., increase in island area) for the three larger Chinese projects was extremely fast (see Fig. 8, Supplementary Fig. 1, and Supplementary Fig. 2 for OLI-based time series of artificial islands creation on Fiery Cross Reef, Mischief Reef, and Subi Reef, respectively). Combined, these new artificial islands and additions to existing islands were measured to be 15.12 km^2^ in area, while individual islands ranged from 0.03 (Central Reef) to 6.39 km^2^ (Mischief Reef). Table 1 summarizes the island building activities as observed from OLI data.

### TI and cloudmask performance

In comparing TI imagery to true color RGB composites, we found that the TI products effectively distinguished clear water from turbidity plumes visible in RGB imagery. Indeed, nearly all turbidity plumes observed in the true color RGB composites were associated with high values in the TI product (Fig. 6). Similar swaths of elevated TI values were also observed in locations where turbidity plumes would be expected based on temporally adjacent imagery, but for which the RGB composites showed no plume. This was especially true for instances of severe sunglint contamination (Fig. 6, left column).

While thin clouds generally presented as low or negative TI, elevated TI values were also often observed near cloud edges or for targets including very small clouds (i.e., not associated with turbidity plumes; see Fig. 6, third column). These false positives primarily indicate a failure of the cloudmasking procedure. Submerged coral reef targets (those not within a turbidity plume) and land pixels (including the artificial islands) showed negative TI, with the latter being much lower. Due to the brightness of the artificial islands, the cloudmask often incorrectly determined such pixels to be cloud. In cases where such cloudmask failures obscured pixels in obvious absence of clouds, manual delineations were used to override the cloudmask.

### Plume detection using MODIS/A

For most dates, the spatial resolution of MODIS/A data was too coarse to confidently distinguish submerged reef from artificial islands using RGB data (Fig. 6). Nevertheless, RGB imagery coupled with the TI products made it relatively easy to delineate turbidity plumes related to island building activities. Plumes were manually delineated using data from January 2013 to December 2015 for Mischief, Subi, and Fiery Cross reefs (turbidity plumes associated with the other island building activities were too small to quantify in this manner). Over this three-year span, the total number of images for which plume extent (if present) could be detected for these three reefs was 205, 129, and 105, respectively, with turbidity plumes identified in about one third of images (70, 43, and 45 images, respectively).

**Figure 9:**
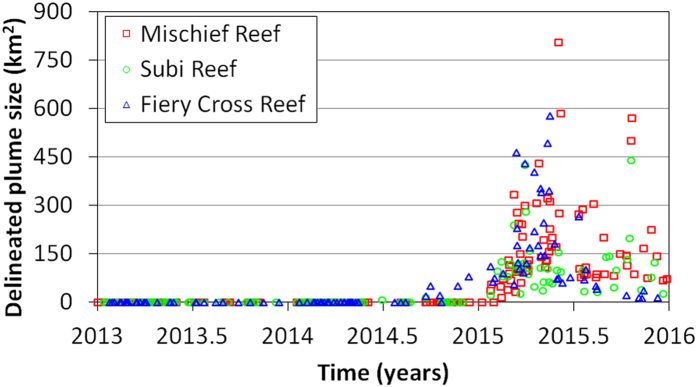
Time series of plume sizes (in km^2^) delineated from MODIS data for Mischief Reef (red squares), Subi Reef (green circles) and Fiery Cross Reef (blue triangles).

For each of the three largest island building projects, turbidity plumes delineated in MODIS/A data began within days of the first OLI observation of island building. Similarly, with a few notable exceptions, the largest turbidity plumes also ceased concurrently with the OLI-observed ends of major island building activities. Construction on these islands was continuous, as plume absence was detected after island building began on only four occasions (combined across these three projects). Time series showing the areal extent of turbidity plumes are compiled in Fig. 9.

**Figure 10:**
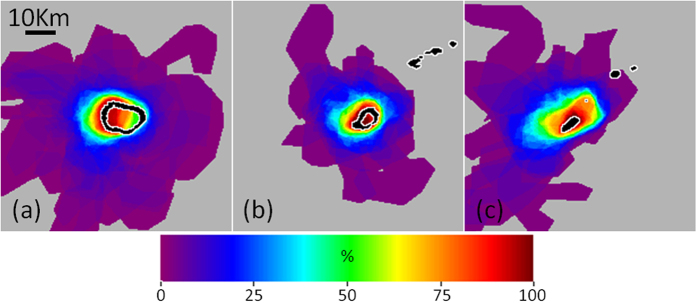
Spatial frequency of delineated plumes (as percent of total) for (**a**) Mischief Reef, (**b**) Subi Reef, and (**c**) Fiery Cross Reef. Coral reef locations indicated in black with white outline.

Within each reef region, the spatial frequency of delineated plumes (Fig. 10) was highest near the location where construction began (as determined from the OLI time series). In total, the spatial extents of delineated plumes for Mischief, Subi, and Fiery Cross reefs were 2,135, 842, and 1,404 km^2^, respectively. Approximately 40% of this area, however, was only impacted once during the time series. Although corals within these particular reefs/atolls were certainly affected by the island building (either through increased turbidity, elevated sedimentation, or physical burial in conversion to islands), we observed no plumes reaching adjacent reefs/atolls.

## Discussion

Together, these results show clear applicability of Landsat-8 OLI and MODIS/A in monitoring and assessing island building activities in the SCS. Although OLI spatial resolution is coarser than that of most commercial satellites, it is nevertheless sufficient to capture these newly created artificial islands. The spatial resolution of MODIS, however, is inadequate to regularly differentiate submerged reef from land using RGB data, especially for large viewing angles or when the submerged reef is overlain by extremely turbid waters (see Fig. 6). Instead, the larger swath and near-daily repeat sampling frequency of MODIS are more adept for identifying turbidity plumes.

Overall, these analyses paint a picture of wholesale environmental changes for many locations in the Spratly Islands. Combined, over 15 km^2^ of land area was created within fifteen previously submerged reefs in a span of under 3 years. For comparison, this is nearly twice the area of the Palm Jumeirah artificial island complex offshore Dubai, United Arab Emirates (8.7 km^2^, estimated using OLI data). All of this land area was built on coral reefs, thus this change represents ecosystem destruction via burial on a truly massive scale. Coral communities nearby the island building activities (i.e., those not converted to land area) also likely suffered from the construction activities^18^,^19^. Although there are no known *in situ* surveys directly documenting the impacts of these specific projects, much smaller turbidity plumes resulting from the 2014 Port of Miami (Florida, USA) dredging^14^ caused massive sedimentation, scour, and burial of coral reefs^20^,^21^. Furthermore, scattering of light by suspended sediments in the water column certainly decreased the light available at depth, likely diminishing the photosynthetic activities of symbiotic zooxanthellae within coral tissues^18^,^19^.

As has been reported in similar turbidity plume studies (e.g., ref. 14), these plumes were observed to be extremely ephemeral, with large day-to-day variability in both size and spatial extent (Fig. 6). Much of this variability is likely due to changes in prevailing currents, combined with rapid settlement of the suspended sediments. Increased temporal resolution of satellite measurements might provide additional insight on the fate of these turbidity plumes. However, due to orbital characteristics, same-day overlap between OLI and MODIS/A data never occurs in this region. Combined with the day-to-day variability in plumes, this lack of overlap also limits direct comparisons between plume size as estimated from MODIS/A and OLI. The MODIS instrument on the satellite Terra might be used to expand the MODIS/A time series, however striping and scan-mirror damage on MODIS/T limit its applications for ocean color research^22^. Ultimately, a geostationary sensor may be launched in the future to cover this region, enabling hourly measurements of turbidity plumes and estimates of the plume advection and settling speeds.

### Detection of artificial islands using MODIS

Despite the capability of monitoring island building activities through visual inspection of OLI imagery, detection of new artificial islands (without *a priori* knowledge of the location, e.g., from media coverage) is more challenging. While OLI includes NIR bands that might be used in a manner similar to the MODIS/A approach, effective cloud (and cloud shadow) masking over water targets is currently problematic. This is especially true in instances of sunglint contamination, which is persistent for much of the year in imagery over the tropical SCS.

Due to the limited spatial resolution of MODIS/A and cloudmasking errors, it is similarly difficult to robustly identify newly created artificial islands (especially small ones) using data from only a single satellite pass. The time-series based approach to artificial island detection removes guesswork from such determinations, allowing for easy detection of new artificial islands (even those affecting only a few pixels) based on *R*_*rc*_(859) trends. Note that for two of the false negatives in Fig. 7 (Sand Cay and Central Reef), construction began prior to the OLI time series (mid-2013). Thus, these omissions would likely be remedied if a longer time series of measurements were included in the slope metric. However, these and the remaining island building activities which were not detected using this method (Pearson Reef, Itu Aba Island, the eastern portion of Cornwallis South Reef) represent only small increases in land area. As such, it is likely that island building activities sized less than 0.06 km^2^ (Table 1) cannot be detected using this method. This size roughly corresponds to the areal extent of a 250 m pixel. No deep-water pixels were identified as being converted to artificial islands, and all identified pixels were corroborated by OLI observations of island building. Thus we infer no false positives with this MODIS/A-based artificial island detection metric. However, the MODIS/A 859 nm band has 250 m spatial resolution at nadir and much coarser resolution nearer the scan edge. As a result, the areal extent of pixels identified using this metric can be larger than that observed with higher resolution sensors, highlighting the primary utility of this metric as a method in directing and informing subsequent analyses.

### Detection of artificial islands using OLI

While very little size documentation exists for most of the artificial island projects assessed in this study, the OLI-based size estimates detailed here compare well with those previously reported for China’s seven island building activities in the SCS^4^,^5^. Despite a substantially different methodology from the OLI-based approach of Mora *et al*.^5^ (whose analysis includes data up to April 2015), reef and island size estimates reported here are quite similar. Nevertheless, the current results show slightly smaller island area (average of 0.06 km^2^ per island and 2.7% unbiased percent difference [UPD]) and smaller reef area (average = 0.2 km^2^ per reef, UPD = 7.5%) than that reported by Mora *et al*.^5^. As such, this portion of the current study reaffirms their general findings, while updating results through early 2016 and incorporating data from smaller island building projects in the SCS.

Contrastingly, the artificial island sizes reported here are larger than those calculated from high-resolution commercial satellites by the Asia Maritime Transparency Initiative (AMTI^4^; average = 0.24 km^2^ per island, UPD = 14.5%). This size discrepancy, however, is much less for the four smaller islands (average = 0.02 km^2^) than the three larger ones (average = 0.53 km^2^). It is possible that the relatively coarse OLI resolution leads to overestimation of these island sizes. In particular, mixed pixels (i.e., 30 m^2^ targets which include reflectance from both artificial island and adjacent non-land sub-pixels) may be preferentially classified as artificial island-only pixels. In addition, the relative brightness of new artificial island targets may influence the satellite measured radiance of nearby darker targets. Indeed, the difference between size estimates from the current study and those reported by AMTI^4^ is strongly correlated to artificial island circumference (as well as artificial island area).

In a similar way, the relatively coarse spatial resolution of OLI data also limits the certainty with which smaller artificial island projects can be quantified. Indeed, it was often difficult to distinguish changes in small artificial islands from naturally occurring changes in exposed land (e.g., due to tide at the time of the satellite measurements, or due to natural sand accumulations). For example, note the change in the western landmass on Central Reef (Fig. 4, #14), for which we can find no reports of island building activities. From this image pair alone, changes in the western landmass appear to be on the same scale as the island building activities documented by the AMTI^4^ on the eastern landmass. In such cases, the time series of OLI data (showing characteristic progression of island building) combined with media reports are often needed to quantify the artificial island size. Similarly, the exact dates for the start and completion of the smallest island building projects (Pearson Reef and Central Reef) were very difficult to definitely identify, further indicating the minimum size of island building activities that can be detected using OLI data in this manner is approximately 0.01 km^2^ (roughly 10 OLI pixels).

### TI algorithm performance and uncertainties

A number of algorithms have been developed by the research community to quantify in-water turbidity [i.e., suspended particulate matter (SPM), total suspended matter (TSM), or total suspended solids (TSS)] from satellite reflectance data. Generally, these approaches use empirical relationships between measured SPM and either red band reflectance^8^,^23^,^24^,^25^,^26^,^27^,^28^ or reflectance ratios between red and green bands^29^. As noted above (Section 2.2., Fig. 5), red band *R*_*rs*_ and *R*_*rc*_ alone were deemed insufficient to distinguish clear water from turbidity plumes in this study. Red/green reflectance ratios also suffered from similar complications, necessitating development of the TI for detection of turbidity plumes resulting from these island building activities.

As a consequence, without prior empirical conversion to SPM and with no *in situ* measurements available for validation, it is impossible to quantify in-water sediment loads in absolute units from TI data. Nevertheless, we compared TI values to those from the *R*_*rs*_(645)-based empirical algorithm developed by Chen *et al*.^30^. Although the relationship between these two metrics is not particularly strong, we estimate that the upper end of the TI colorbars (Figs 5 and 6) represent SPM values on the order of 8–10 nephelometric turbidity units (NTU).

Despite this lack of validation, TI data can be used to qualitatively assess turbidity under a suite of viewing conditions, even those which mask the *R*_*rs*_ data that form the basis of traditional turbidity algorithms. Indeed, the first column in Fig. 6 demonstrates detection of plumes (even those not detectable in the true-color RGB imagery) under severe glint conditions (these pixels are identified as HIGLINT by the Level 2 processing flags; see ref. 17). Note, however, that this glint correction is not without error. The coefficients listed for Eq. 2 were derived to be applicable for any pixel (globally), yet a variety of factors (e.g., windspeed and atmospheric conditions) may influence the optimal coefficients for a particular date or location. This is especially true for the blue bands, while coefficients for the red bands are comparatively more stable. Since the TI is based on *R*_*rc*_ differences between the blue and red bands, the spectral component of this uncertainty for sunglint-affected pixels can compromise the robustness of individual TI values. However, these uncertainties generally manifested equally (or in a smooth gradient) across a scene, making them easy to detect and dismiss during manual plume delineations.

Apart from the minor glint-related uncertainties, background TI values are not completely stable between images (notice slight color differences for non-plume waters between panels in Fig. 5e,j, as well as in the bottom row of Fig. 6). These are likely due to uncertainties in the correction for ozone and gaseous absorption as well as changing aerosols for different images, and serve to justify the primarily qualitative uses for TI in plume delineation. Furthermore, errors in masking of cloud edges and thin clouds often presented as elevated TI values in the MODIS/A imagery (see Fig. 6, bottom row). However, these errors were smaller and easily distinguishable from turbidity plumes, and thus are generally excluded from the manual plume delineations.

Given the uncertainty sources listed above, manually delineated turbidity plumes are imprecise. The size and shape of delineations can be greatly affected by a variety of factors, including the specific color ramp, the data scaling, plume intensity, observer viewing conditions, or inter-observer biases. While the latter is not relevant here (delineations were all made by a single observer), these, and other, sources of error lead to potentially large uncertainties. It is important to note, however, that a pessimistic estimate of 50% uncertainty does not change the overall conclusion of massive turbidity plumes from these island building activities that have no observed historical analog in the study region. Thus, the conclusion of significant changes in turbidity plumes due to island building is valid regardless of all these complicating factors.

### Future perspective

Under a changing climate, human activities have been shown to play a critical role in reshaping environments. Dam construction, port dredging, and artificial island building can all alter the environment both directly and indirectly. How to detect, monitor, and assess the environmental changes in the vast ocean, especially over remote regions or regions of intense geopolitical conflict, has remained a technical challenge. In this regard, the case study here not only provides comprehensive information on environmental changes in the 15 specific locations of the SCS, but also (and more importantly) shows a technical approach to address such a challenge using publicly available satellite remote sensing data. Such an approach, once implemented for the entire SCS or other similar ocean environments (e.g., the Paracel Islands), may provide a fast and effective means to assist environmental stewardship for better planning and management. With the new satellite sensors being planned for the near future by both NASA and the European Space Agency, such a capacity is expected to be enhanced significantly.

## Conclusions

The nature of island building activities in the Spratly Islands, particularly the widespread ecological damage, warrants scientific attention through quantitative assessment and monitoring. Given the scale and remoteness, such research requires measurement approaches which are synoptic, frequent, and (per geopolitical tensions) non-intrusive. In this work, we demonstrated that freely available Landsat-8 OLI and MODIS/A data from satellite remote sensing platforms can be used for detection and quantification of artificial island creation and associated turbidity plumes. The regular repeat sampling by these sensors allowed for evaluation of changes relative to a historical context, and highlighted the time sequence of environmental shifts. In doing so, massive alterations to shallow coral ecosystems, through direct burial and indirect effects of turbidity plumes, have been documented. As such activities in the SCS (and elsewhere) are likely to continue, we recommend implementation of these approaches for the entire SCS toward more robust environmental assessment and monitoring of future artificial island creation.

## Additional Information

**How to cite this article**: Barnes, B. B. and Hu, C. Island building in the South China Sea: detection of turbidity plumes and artificial islands using Landsat and MODIS data. *Sci. Rep.* **6**, 33194; doi: 10.1038/srep33194 (2016).

## Supplementary Material

Supplementary Information

## Figures and Tables

**Table 1:** Summary of island building activities observed in OLI time series.

Reef (Country performing construction*)	Latitude (N)	Longitude (E)	Reef area (km^2^)	Island area (km^2^) (new area)†	Approx. start of island building	Approx. end of island building
Mischief Reef (C)	9.90	115.54	13.8	6.39	Jan 2015	Dec 2015
Subi Reef (C)	10.93	114.08	7.12	4.43	Feb 2015	Nov 2015
Fiery Cross Reef (C)	9.55	112.89	4.41	3.06	Aug 2014	June 2015
Cuarteron Reef (C)	8.86	112.83	5.07	0.25	Mar 2014	Dec 2014
Gaven Reef (C)	10.21	114.22	1.83	0.19	May 2014	Jan 2015
Johnson South Reef (C)	9.71	114.29	6.81	0.11	Feb 2014	Jul 2014
Hughes Reef (C)	9.91	114.50	2.59	0.09	Apr 2014	Jun 2014
Spratly Island (V)	8.65	111.92	0.45	0.33† (0.17)	Feb 2015	Ongoing
Sin Cowe Island (V)	9.88	114.33	1.09	0.17† (0.11)	Apr 2014	Feb 2015
Cornwallis South Reef (V)	8.69	114.19	14.98	0.12	Apr 2015	Ongoing
West Reef (V)	8.87	112.25	0.61	0.10	Pre-OLI	Dec 2015
Sand Cay (V)	10.38	114.48	1.18	0.07† (0.02)	Pre-OLI	Nov 2013
Pearson Reef (V)	8.98	113.71	10.32	0.04† (<0.01)	Feb 2015	Mar 2016
Central Reef (V)	8.93	112.35	0.97	0.03† (<0.01)	Pre-OLI	Dec 2015
Itu Aba Island (T)	10.38	114.37	1.57	0.49† (0.06)	Mar 2014	Oct 2015

Total new island areas reported here are 14.52 km^2^ (China), 0.54 km^2^ (Vietnam), and 0.06 km^2^ (Taiwan).

^*^Does not imply territorial ownership. C = China, V = Vietnam, T = Taiwan.

^†^Recent construction activities for these islands expanded previously existing natural or artificial islands. Area reported in parentheses is that added during the OLI time series.
