# Supplementary material for: Island building in the South China Sea: detection of turbidity plumes and artificial islands using Landsat and MODIS data
Source: Sci Rep. 2016 Sep 15;6:33194. doi: 10.1038/srep33194 (PMC5024115; doi:10.1038/srep33194)
Supplement: Supplementary Information [file srep33194-s1.pdf]

Supplementary material for manuscript submitted to *Scientific Reports:*

Island building in the South China Sea: detection of turbidity plumes and artificial islands using Landsat and MODIS data

Brian B. Barnes*, Chuanmin Hu

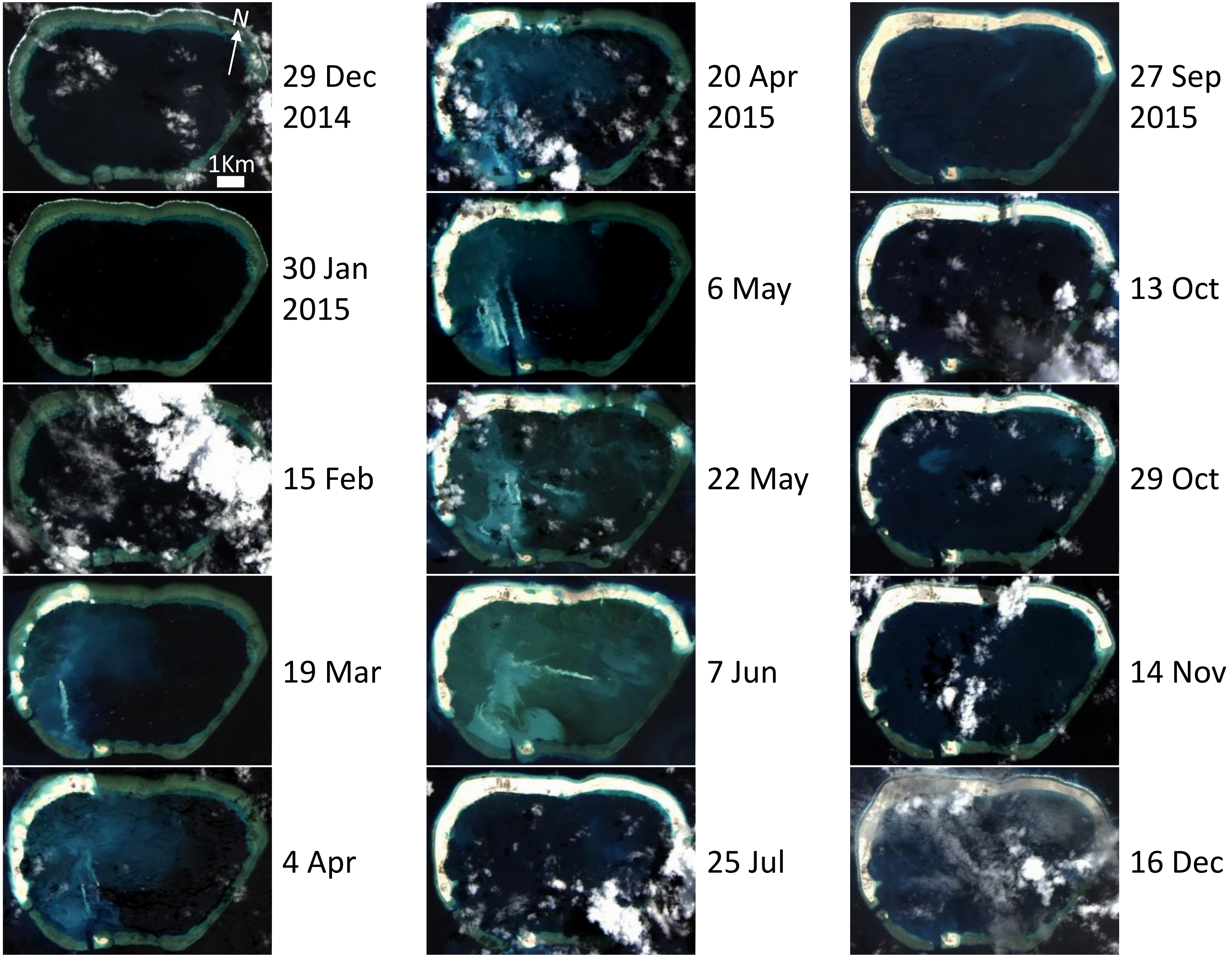
N
1Km
29 Dec 2014
30 Jan 2015
15 Feb
19 Mar
4 Apr
20 Apr 2015
6 May
22 May
7 Jun
25 Jul
27 Sep 2015
13 Oct
29 Oct
14 Nov
16 Dec

Figure S1

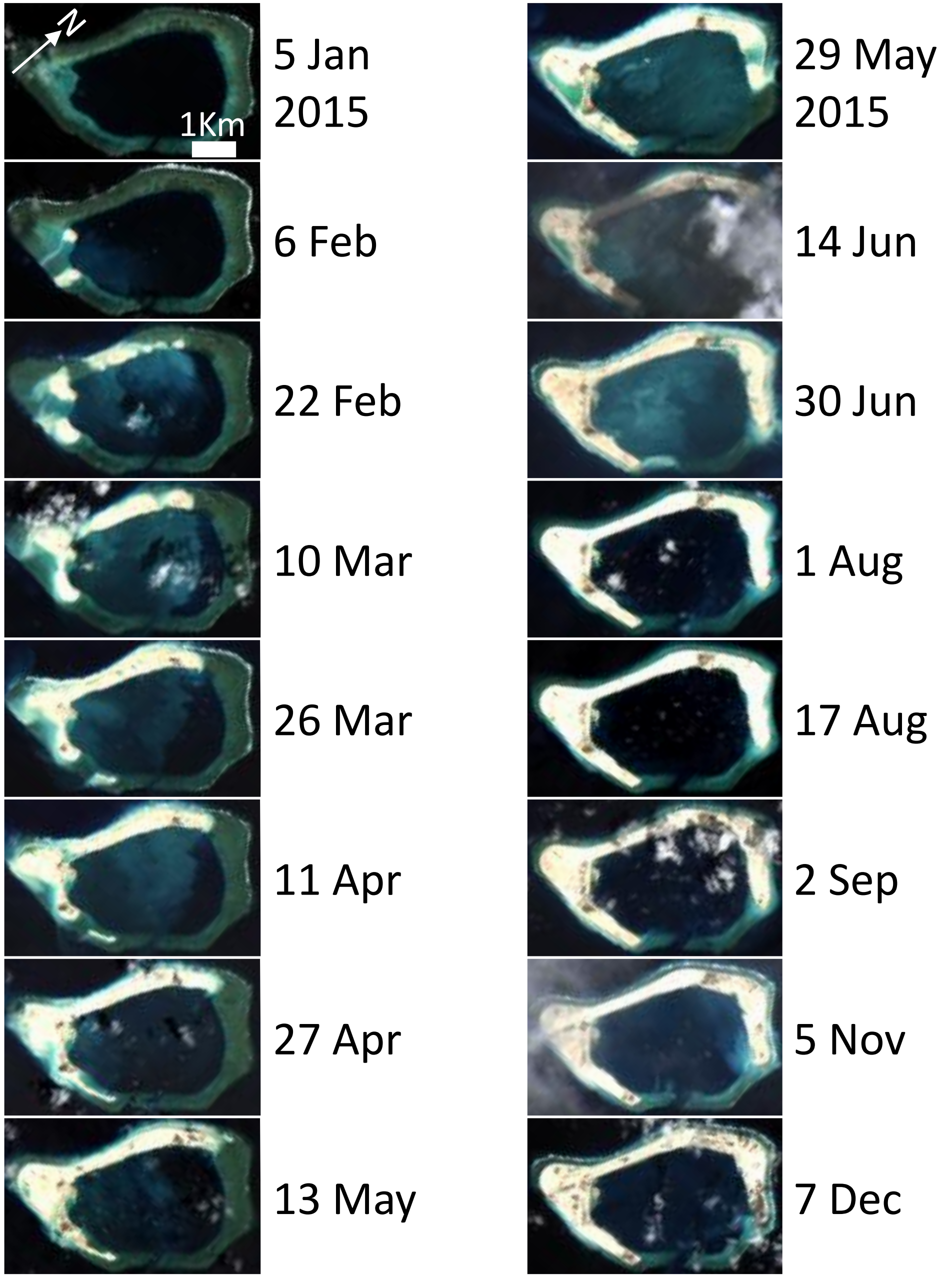
N
1Km
5 Jan 2015
6 Feb
22 Feb
10 Mar
26 Mar
11 Apr
27 Apr
13 May
29 May 2015
14 Jun
30 Jun
1 Aug
17 Aug
2 Sep
5 Nov
7 Dec

Figure S2

Supplemental figure legend

Figure S1: Time series of island building for Mischief Reef from Landsat-8 OLI. These images were created using data from the USGS (http://earthexplorer.usgs.gov/) and processed using Acolite (version 20150701.1) as detailed in the methods section.

Figure S2: Time series of island building for Subi Reef from Landsat-8 OLI. These images were created using data from the USGS (http://earthexplorer.usgs.gov/) and processed using Acolite (version 20150701.1) as detailed in the methods section.
